# Chest computed tomography (CT) findings and semiquantitative scoring of 60 patients with coronavirus disease 2019 (COVID-19): A retrospective imaging analysis combining anatomy and pathology

**DOI:** 10.1371/journal.pone.0238760

**Published:** 2020-09-04

**Authors:** Hao Zhang, Xu-jing Jiang, Xiao-hua Liu, Hong Ma, Ya-hong Zhang, Yue Rao, Lin Li, Hai-yan Xu, Fa-jin Lyu

**Affiliations:** 1 Department of Radiology, Dianjiang People’s Hospital of Chongqing, Chongqing, China; 2 Department of General Surgery, Dianjiang People’s Hospital of Chongqing, Chongqing, China; 3 Department of Oncology, Dianjiang People’s Hospital of Chongqing, Chongqing, China; 4 Department of Radiology, Changshou People’s Hospital of Chongqing, Chongqing, China; 5 Department of Radiology, Zhongxian People’s Hospital of Chongqing, Chongqing, China; 6 Department of Pharmacy, Dianjiang People’s Hospital of Chongqing, Chongqing, China; 7 Department of Gastroenterology, Dianjiang People’s Hospital of Chongqing, Chongqing, China; 8 Department of Radiology, First Affiliated Hospital of Chongqing Medical University, Chongqing, China; Erasmus MC, NETHERLANDS

## Abstract

In this study, we ascertained the chest CT data of 60 patients admitted to 3 hospitals in Chongqing with confirmed COVID-19. We conducted anatomical and pathological analyses to elucidate the possible reasons for the distribution, morphology, and characteristics of COVID-19 in chest CT. We also shared a semiquantitative scoring of affected lung segments, which was recommended by our local medical association. This scoring system was applied to quantify the severity of the disease. The most frequent imaging findings of COVID-19 were subpleural ground glass opacities and consolidation; there was a significant difference in semiquantitative scores between the early, progressive, and severe stages of the disease. We conclude that the chest CT findings of COVID-19 showed certain characteristics because of the anatomical features of the human body and pathological changes caused by the virus. Therefore, chest CT is a valuable tool for facilitating the diagnosis of COVID-19 and semiquantitative scoring of affected lung segments may further elucidate diagnosis and assessment of disease severity. This will assist healthcare workers in diagnosing COVID-19 and assessing disease severity, facilitate the selection of appropriate treatment options, which is important for reducing the spread of the virus, saving lives, and controlling the pandemic.

## Introduction

In December 2019, cases of pneumonia with unknown cause were confirmed in Wuhan, Hubei province, China and spread rapidly throughout China and the rest of the world [1, 2]. The World Health Organization later named the novel coronavirus responsible for the outbreak as Corona Virus Disease 2019 (COVID-19). It is highly contagious and can cause severe respiratory symptoms leading to death [3]. As of June 30, 2020, >200 countries and regions have been affected with >10.1 million confirmed cases and >503,000 deaths [4]. At present, diagnosis of COVID-19 is confirmed by the real-time reverse transcription polymerase chain reaction (RT-PCR) test, which has high specificity but low sensitivity for diagnosing the disease [5, 6]. This test may lead to missed diagnoses and thus increase the spread of the disease. Chest computed tomography (CT) has been an effective and important supplement to RT-PCR testing in the diagnosis of COVID-19 [7]. However, few correlations of anatomical–pathological–radiological analyses of COVID-19 have been conducted because COVID-19 autopsies are few. However, autopsy findings and related pathological analyses of COVID-19 cases have recently been reported in China [8–11]. The disease predominantly causes inflammatory responses characterized by deep airway and alveolar injury. Therefore, this study had two aims. The first was to describe the chest CT imaging manifestations in 60 patients with COVID-19 using anatomical and pathological analyses, to clarify the value of chest CT in diagnosing COVID-19. The second aim was to share a semiquantitative scoring system used to quantify the severity of COVID-19 pneumonia, which supplements diagnostic information and clinical potential for facilitating treatment decisions.

## Materials and methods

### Ethics

This retrospective study was approved by the ethics committees (Institutional Review Boards of the Diangjiang, Changshou and Zhongxian People’s Hospital [Approval number: DJ20200202, CS20200203 and ZX20200202]) of the three participating hospitals. As this was a retrospective study using archive CT data, the Ethical Committees waived the requirement for written informed consent. All procedures on research were performed in accordance with the relevant guidelines and regulations.

### Study participants

We collected data on 60 consecutive patients admitted to hospital with COVID-19 confirmed by RT-PCR in 3 cities within the jurisdiction of Chongqing municipality, bordering Hubei province, between January 22nd and February 15th, 2020. There were no exclusion criteria.

### CT data acquisition and analysis

We used a multi-slice spiral CT scanner (Siemens Somatom Emotion 16-detector CT, Erlangen, Germany; GE Light Speed and GE Bright Speed 16-detector CT, Milwaukee, Wis) to obtain chest images, without contrast agent injections, at the end of inhalation in the supine position. However, some patients could not cooperate with breath-holding during scanning. The scanning protocols were as follows: 120 kV, 150mA, 5mm slice thickness. Axial images were reconstructed with a 1.0 mm section thickness and a 1.0 mm section interval. We evaluated and recorded imaging features according to the Fleischner Society lexicon [12], recording the presence or absence of the following features: ground glass opacities (GGO), consolidation, reticular pattern, crazy paving pattern, airway changes, pleural changes, fibrosis, nodules, halo sign, subpleural curvilinear line, vascular enlargement, and lymphadenopathy (short axis ≥10 mm). We used a semiquantitative scoring system to quantify the severity of COVID-19. Lung segments were divided into the following using an anatomical reference atlas: S^1^ (apical), S^2^ (anterior), S^3^ (posterior),…, S^10^ (posterior basal). The left and right lungs were divided into 10 segments for comparison and scoring. We then scored each infected lung segment 1 score and 2 scores were given to the lung segments that were infected with volumes of 1%–50% and 51%–100% in the visual measurements. The total score was calculated as the sum of the individual scores in each lung segment, and could range between 0 and 40 points. The intrapulmonary regions were classified as follows: central (mainly in the inner third of the lungs), peripheral (mainly in the outer third of the lungs), and intermediate (between the inner third and the outer third of the lungs). We classified chest CT manifestations of COVID-19 into three stages according to Chinese guidelines: early, progressive, and severe stages [13] (In the early stage, single or multiple GGO with peripheral distribution was the main imaging manifestation. In the progressive stage, there was an increase in infected lung tissues, with reticular and crazy-paving patterns and consolidation. In the severe stage, infections were further aggravated and were diffusely distributed, with apparent dense consolidation). These disease stage classifications were independently reviewed and evaluated by two radiologists with 18 years (Y.H.Z.) and 19 years (H.Z.) experience, respectively. The remaining chest CT data were independently reviewed and evaluated by two radiologists with 8 (X.H.L.) and 4 years (Y.R) of experience. All differences were settled by consensus.

### Statistical analysis

We used SPSS version 26.0 for Windows (SPSS Inc., Chicago, IL, USA) to analyze the data. Continuous data were summarized using the mean and SD or median and interquartile range (IQR). The Shapiro–Wilk test was used to test for normal distribution of continuous variables.

The mean values of the two groups of continuity variables were compared using the independent samples Student t test or the Mann–Whitney U test, as appropriate. The one-way ANOVA or rank sum test of multiple independent samples was used for inter-group comparison of multiple groups (≥3), and the Bonferroni correction was used for post hoc multiple comparisons. Categorical data were summarized using frequencies (proportions), and frequencies were compared using the Pearson chi-square test or the Fisher exact test as appropriate. A value of *P* < .05 was considered statistically significant. In order to better represent and compare the data, we used GraphPad Prism version 8.0 (GraphPad Software, La Jolla, CA, USA) for data visualization.

## Results

**Fig 1:**
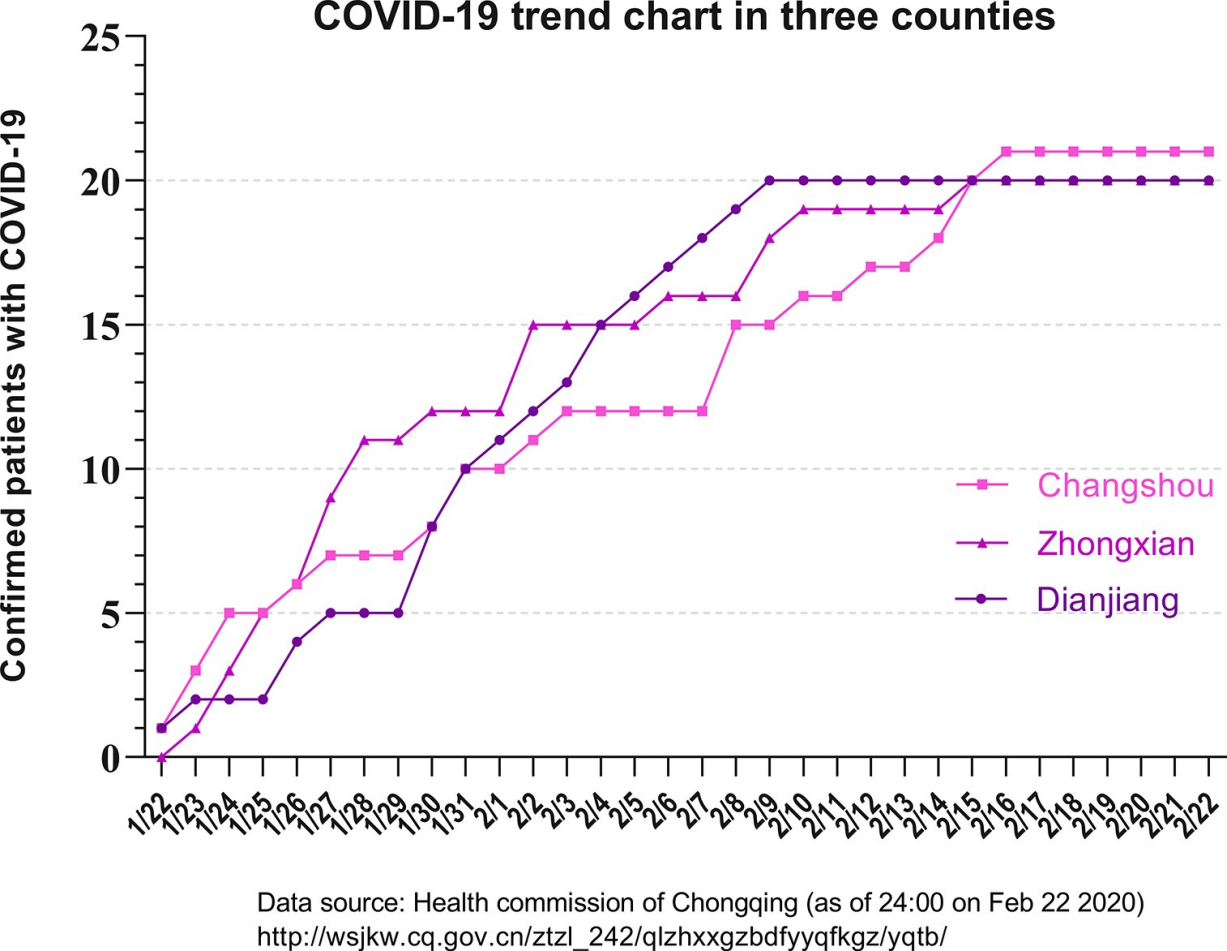
COVID-19 trend chart in three counties. The population of the three counties is similar; each has a total population of between 500 000 and 600 000 and an urban population of approximately 100 000. Between February 16th and May 16th, there were no new COVID-19 patients in Dianjiang, and only 1 and 2 new cases in Zhongxian and Changshou, respectively.

**Fig 2:**
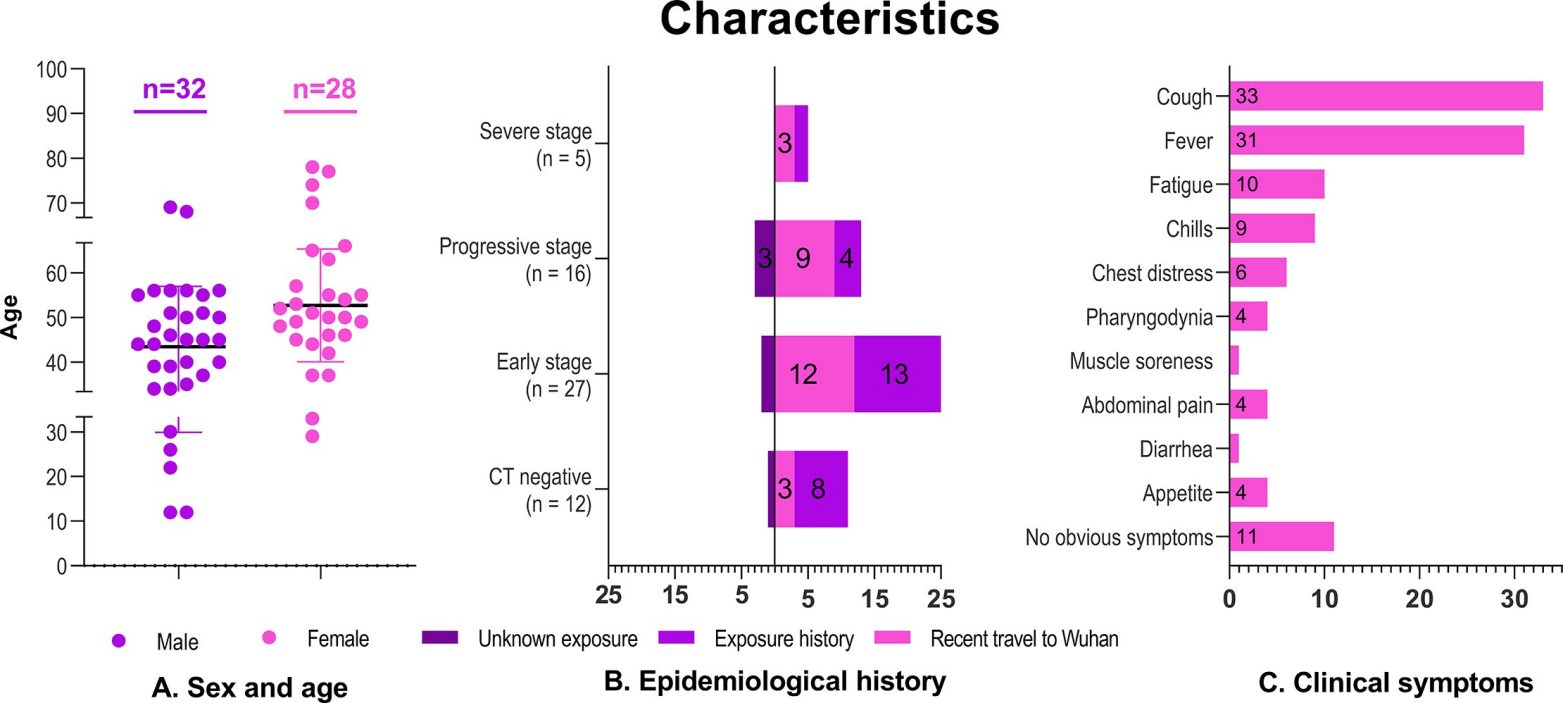
Demographic, epidemiological, and clinical characteristics of the 60 patients with COVID-19. A. There was no significant difference in the mean age between males (43.4, SD 13.5; range 12–69) and females (52, SD 12.6; range 29–78; *P* = .759). B. The numbers of patients with recent travel to Wuhan, exposure history, and unknown exposure history, were 27, 27, and 6, respectively. C. The most common clinical features were cough (33/60) and fever (31/60), and some patients developed gastrointestinal symptoms (9/60).

**Fig 3:**
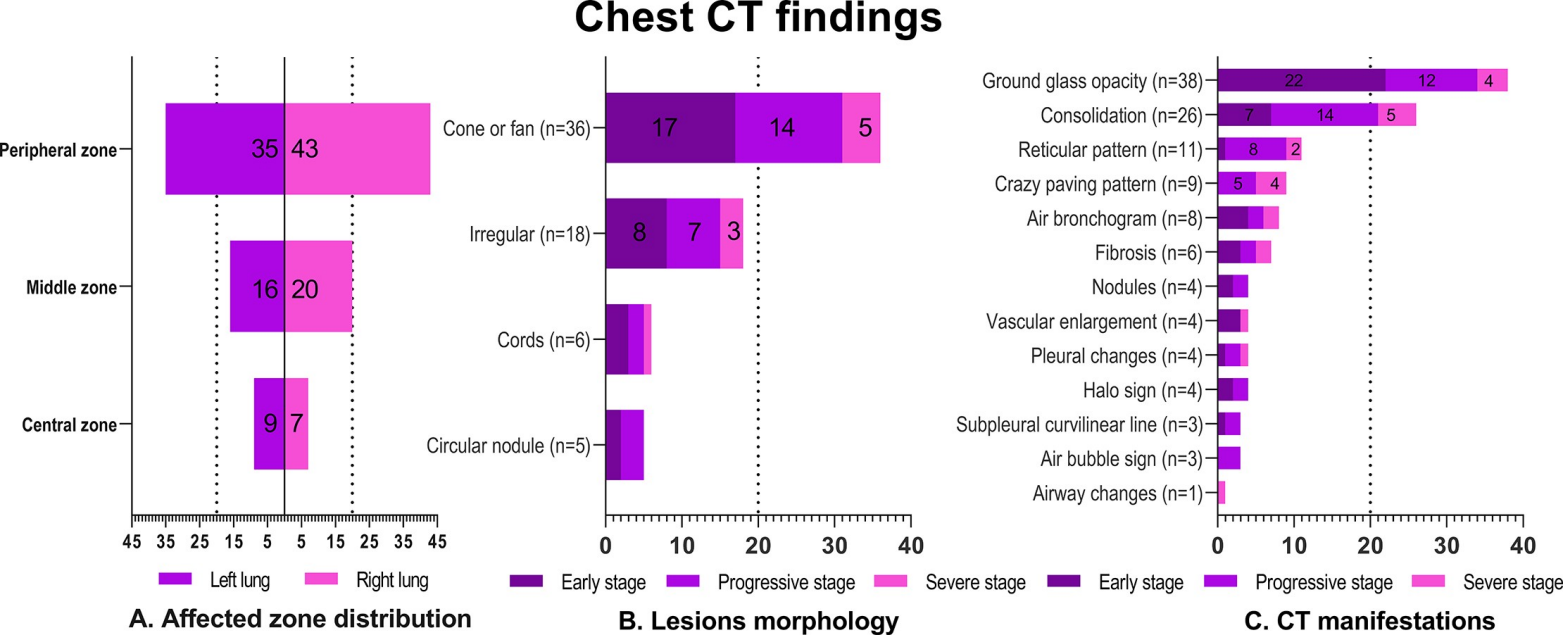
Chest CT findings of the 60 patients with COVID-19. A. There were a significantly greater number of lesions distributed in the peripheral zone compared to the middle zone and the central zone (both *P* < .001). B. The lesions’ morphology was mostly conical or fan (36/48). C. GGO was the most frequent manifestation in the early stages of disease and the most frequent manifestation regardless of disease stage. However, these signs were often coexisted in chest CT.

**Fig 4:**
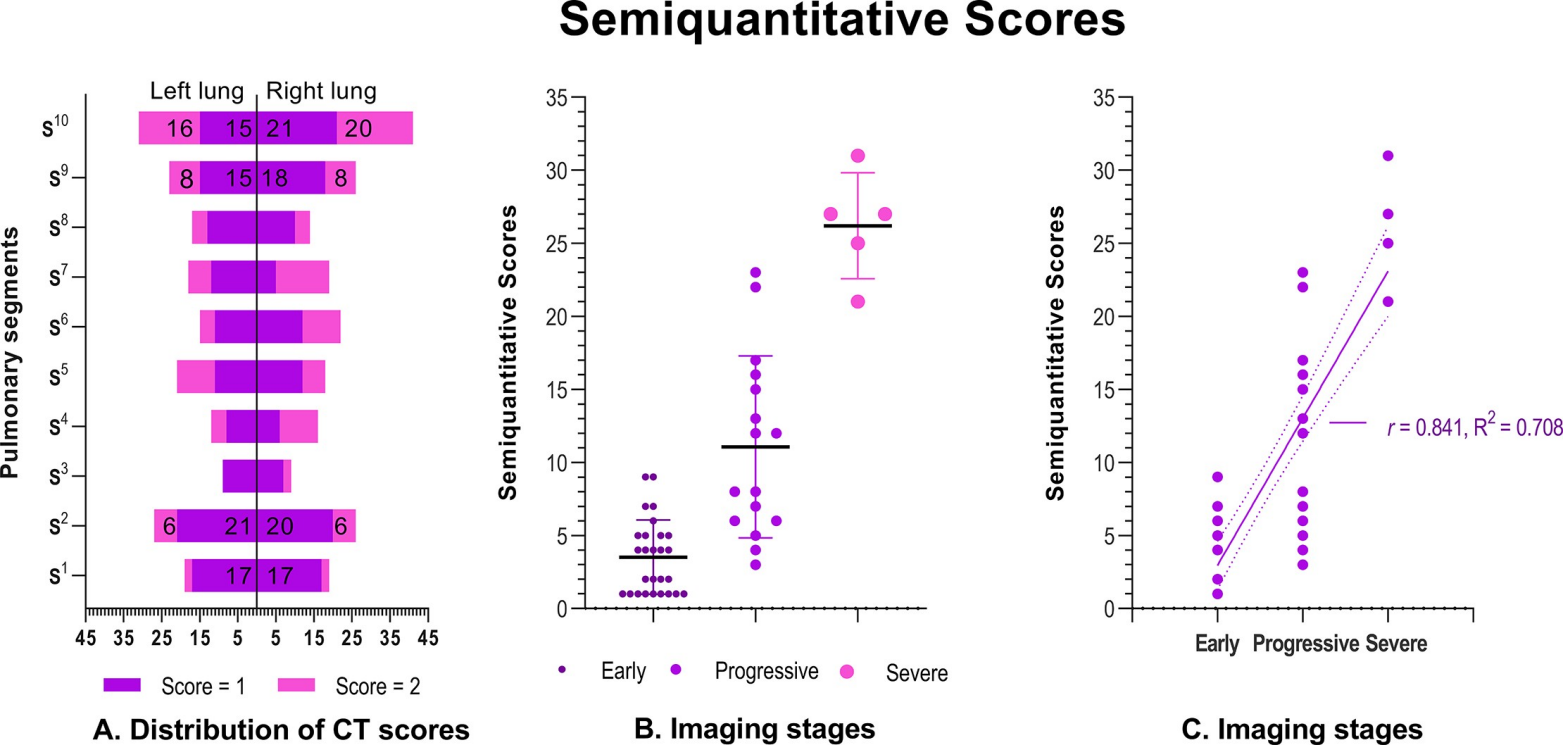
Semiquantitative scores by distribution and imaging stage. A. Lesions were mainly distributed in posterior or near posterior lung segments S^1^, S^2^, S^9,^ and S^10^; semiquantitative scores were highest in S^10^ (posterior basal), especially in the right lower lobe. B. The mean semiquantitative scores were significantly different between the three disease stages; early stage 3.5 (SD 2.5; (1–9), progressive stage 11 (SD 6.2 (3–23), and severe stage 26.2 (SD 3.6 (21–31), *P* < .001. C. There was a significant positive correlation between imaging stage and semiquantitative scores; *r* (correlation coefficient) = 0.841(*P* < .001), R^2^ (goodness of fit) = 0.708.

**Fig 5:**
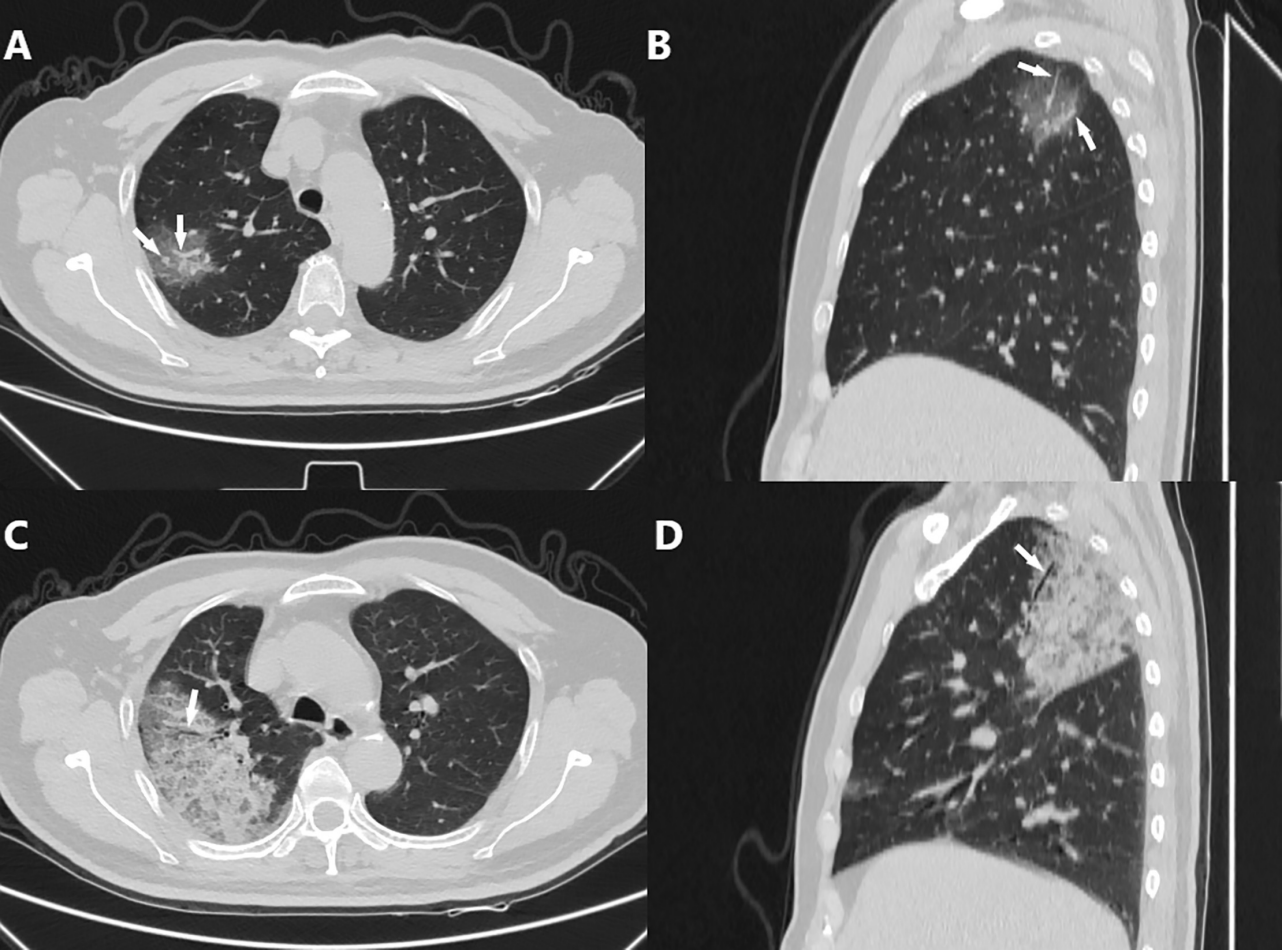
Typical CT manifestations 1. Chest CT images of a 68-year-old male who presented with cough and fever for 2 days. A and B. Axial CT scan (A) and sagittal reconstruction (B) shows a small area of approximately fan-shaped and subpleural GGO in the apical segment of the right upper lobe, with multiple small vascular enlargements (white arrows); the semiquantitative score = 2. C and D. Three days later, the lesion had progressed rapidly, and is displayed as a large area of fan-shaped/conical and subpleural crazy paving pattern with air bronchogram (white arrow); the semiquantitative score = 7.

**Fig 6:**
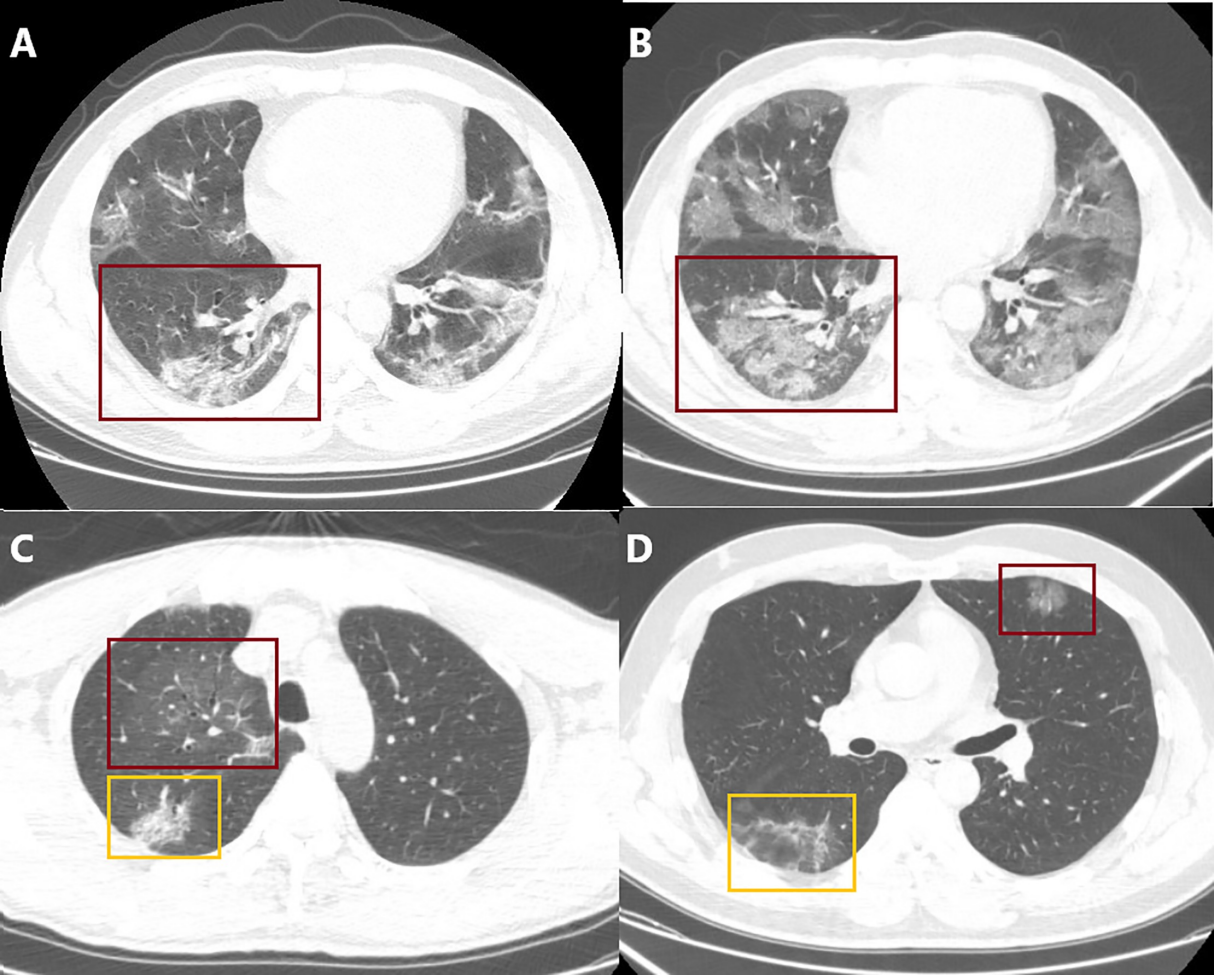
Typical CT manifestations 2. A and B. An initial CT scan (A) compared with a follow-up scan 27 hours later (B) showed that the GGO co-existed with consolidations (red frame) and progressed rapidly. The scope was significantly expanded, and consolidative lesions increased. The initial semiquantitative scores were left lung = 14, and right lung = 13, totaling 27; the follow-up semiquantitative scores were left lung = 19, and right lung = 17, totaling 36. C. The light density of GGO (red frame) with multiple air bronchogram, or more appropriately, bronchiectasis. Subpleural reticular pattern (yellow frame) in the apical segment of the right superior lobe. D. GGO (red frame) and reticular pattern (yellow frame) are located in the subpleural area of the left and right lung, respectively.

**Fig 7:**
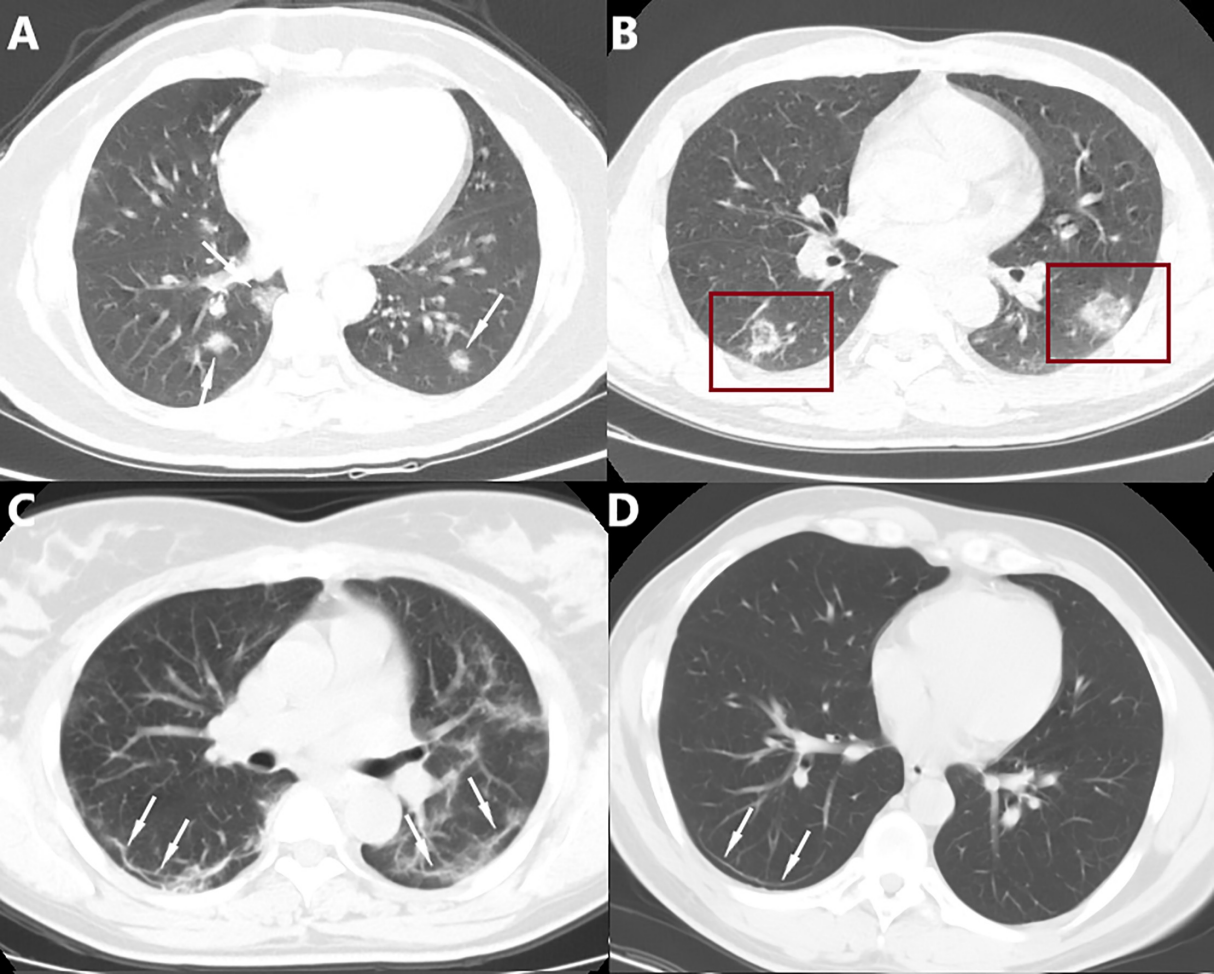
Relatively atypical CT manifestations. A. Multifocal solid irregular nodules (white arrow) with well- or poorly defined edges are shown in the subpleural area of the inferior lobes. B. A reversed halo sign (red frame) was in the subpleural area of the inferior lobes. C. Subpleural curvilinear opacities with poorly defined edges paralleling the pleural surface are shown in the inferior lobes. D. A subpleural thin curvilinear opacity with well-defined edges paralleling the pleural surface in the inferior lobe.

**Fig 8:**
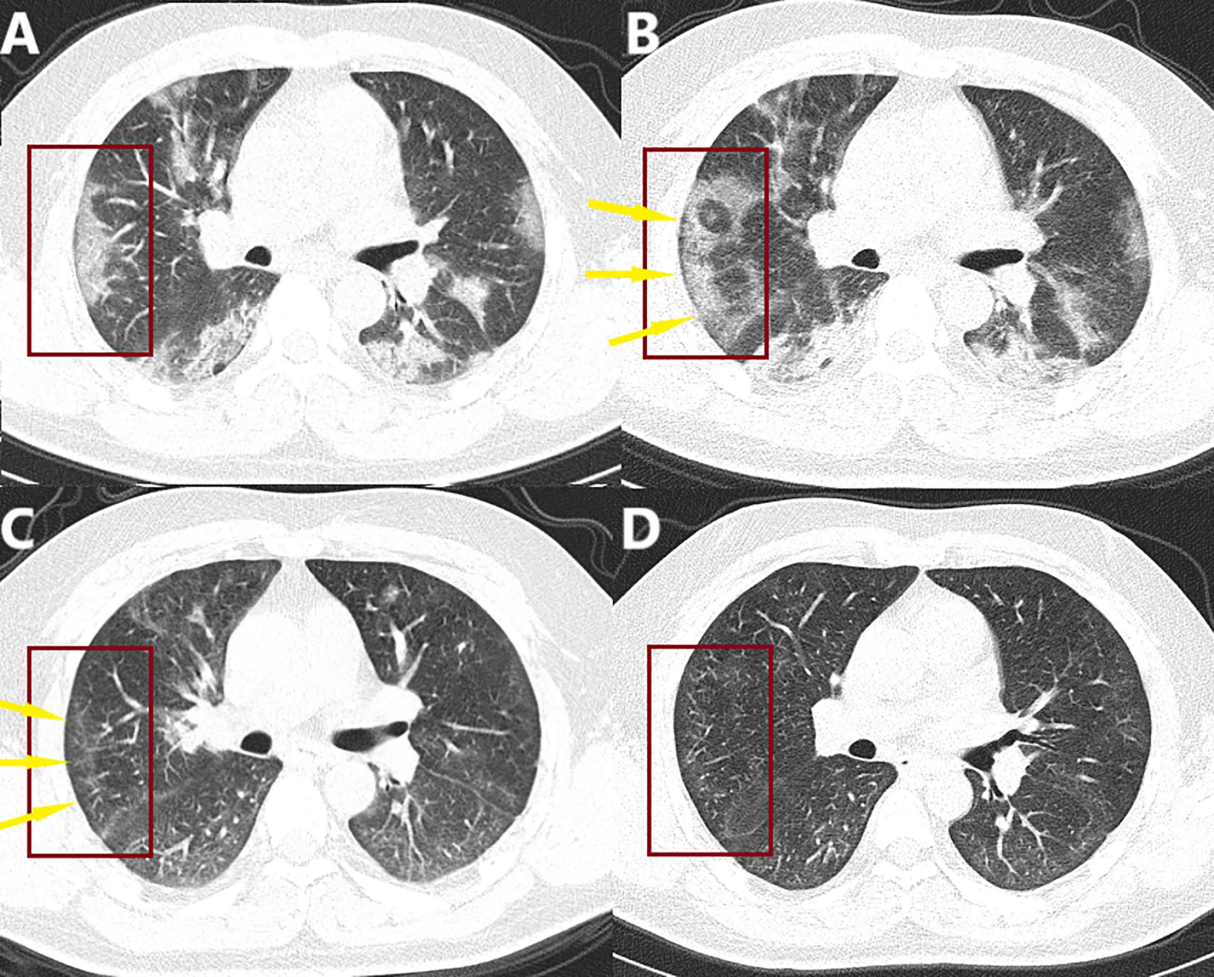
A 34-year-old male who presented with fever for 6 days and cough for 2 days. A. January 21, 2020. The patient’s initial CT scan showed multifocal subpleural conical GGO, locally with consolidation. The patient was diagnosed as severe by chest CT with the semiquantitative score of 25. B. January 26, 2020. The range of lesions (red frame) was enlarged but the density decreased, with a subpleural thin curvilinear lucency (yellow arrow) paralleling the pleural surface, indicating that the lesion is beginning to be absorbed. C. February 26, 2020. The patient’s post-discharge CT review confirmed the initial observation was correct; the lesions were markedly reduced, and the subpleural thin curvilinear lucency (yellow arrow) is still visible. D. March 12, 2020. On reexamination, the lesions had reduced further. However, pulmonary hypoventilation caused by lung tissue damage was also seen, shown as an uneven increase in lung tissue density due to increased X-ray attenuation.

A total of 60 patients with COVID-19 were enrolled, including 20 in Dianjiang, Changshou and Zhongxian (Fig 1). The demographic, epidemiological, and clinical characteristics of the 60 patients are shown in Fig 2. Follow-up scans were obtained in only ten cases. All the patients recovered and were discharged without death after hospitalization. All of the infected persons with exposure history (27/60) were family members of patients who had recently traveled to Wuhan (27/60). A 35-year-old male patient (positive chest CT, early stage) in Dianjiang infected 9 family members, 3 were negative chest CT and 6 were early stage (positive chest CT). The chest CT findings and semiquantitative scores of the affected lung segments are shown in Figs 3 and 4, respectively. Typical and relatively atypical CT manifestations are shown in Figs 5–8.

## Discussion

Only 5/60 (8.3%) patients in this study had severe stage disease. This may be related to the government’s measures to actively respond to COVID-19; patients with suspected COVID-19 underwent both RT-PCR testing and chest CT (possibly multiple times as required), and all persons with a history of exposure to suspected COVID-19 patients were isolated for observation. Therefore, it is likely that the COVID-19 cases in the three cities in this study were predominantly imported cases that were then largely transmitted through family aggregation.

Similar imaging findings but different interesting views have been reported on chest CT studies of COVID-19 as the epidemic evolves. However, we have noted that few correlations of anatomical–pathological–radiological analyses have been conducted and that in-depth discussion on the formation of imaging features is lacking. Recently, autopsy findings and pathological analyses of COVID-19 cases were reported in China [8–11]. The pathological changes in the lung were as a result of inflammatory responses with deep airway and alveolar epithelial injuries, and direct virus attack and immune damage coexisted. Based on these autopsy results and studies, anatomical and pathophysiological considerations were used to analyze the current set of CT chest scans. In our opinion, pathology is the observation of microanatomy, whereas radiology is the observation of gross anatomy. Based on the correlation between pathology and radiology as well as based on relevant research, discussing the internal relationship between anatomy, pathology, and imaging findings should be an additional feasible way of learning. The predominant distribution of COVID-19 infection is subpleural and of the lower lobes, which may be due to the inherent anatomy of the lungs. The conceptual and anatomical differences between the peripheral cortex and the central medulla in the lung region have been proposed by several authors [14, 15]. The peripheral pulmonary cortex consists of 2–3 rows of well-defined secondary pulmonary lobules, forming conical or fan-shaped shapes approximately 3–4cm thick on the outer periphery of the lung or the adjacent cortical pulmonary surfaces of the interlobular fissures. Lobules are the basic unit of the anatomical structure and comprise 3–5 terminal bronchioles and their distal lung tissues. The interlobular septa are multilateral with a size of 1–2.5cm. The normal interlobular septa are not visible on CT [16]. Virus particles are inhaled through the respiratory tract, the main route of invasion, and reach deeper into the lungs, into the lobules of the peripheral pulmonary cortex. The posterior part of the lung has more peripheral pulmonary cortical lobules than the anterior part, which means that bronchioles are more numerous and distributed [17], leading to a greater probability of infection by inhaled virus particles. Furthermore, the anatomical features of the right lower lobe bronchi are relatively straight and steep, increasing the probability of infection beginning in the lobes [18].

Virus particles enter the cell via angiotensin-converting enzyme-2 (ACE2) receptors, which are expressed mainly in type II alveolar epithelia on the endothelial surface [19]. The human immune response is triggered, and the virus is subsequently attacked by clustered immune cells. Thus, the type II alveolar epithelium suffered both direct virus attack and collateral immune damage. Subsequently, alveolar walls are injury edema and thickening, alveolar epithelial hyperplasia, inflammatory cell infiltration (mainly macrophages and monocytes), capillaries expansion hyperemia, and air-filled alveolar space are compressed [9, 18]. These pathological changes resulted in increased density and decreased air content in the area of the lesions. The linear attenuation coefficient of the X-ray is proportional to the material density; therefore, these pathological changes are shown as GGO with pulmonary acinus and/or secondary lobule as the basic unit in chest CT. The lesions are mostly conical or fan-shaped (namely the peripheral/subpleural cortex lobular morphology), forming the early imaging findings.

As the course of pneumonia progresses, multiple lobules expand by or undergo fusion; interstitial inflammatory cells infiltration and viral cytopathic effect aggravate [10]; the alveolar wall significantly thickens; pulmonary interstitial edema is observed; and the exudate in the alveolar cavity increases to varying degrees, led to X-ray attenuation of lesions is enhanced compared with the early. This pathological pattern, primarily characterized by alveolar epithelial damage, followed by pulmonary interstitial edema, with subsequent changes in alveolar space with respect to shape and density, resulting in lesions observed on chest CT performed for consolidation, showing reticular or crazy paving pattern. These were common imaging signs observed during the progression of COVID-19, second only to GGO. Crazy paving pattern has a similar pathological basis to reticular pattern, but it is more severe in degree and is also a signal that pneumonia is entering its peak stage [20].

In the severe disease stage, the following features were observed: diffuse alveolar damage with fiber myxoid exudate; hyaline membrane formation in the alveoli; destruction of alveolar walls with focal hemorrhage; hyaline thrombus formation in microvascular vessels; significantly denatured proliferated type II alveolar epithelia; and necrotic shedding and mucous composition in the bronchioles, resulting in airway obstruction, and air-blood barrier damage. The clinical manifestation of these features is acute respiratory distress syndrome [8, 19]. These pathological changes led to further increases in X-ray attenuation, and chest CT scans showed large areas of GGO, reticular pattern or crazy paving pattern and consolidation.

If the patient recovers, the lesions under the pleura are usually the first to clear, forming a subpleural thin curvilinear lucency paralleling the pleural surface that approximate the density of normal lung tissue. This is because the pulmonary cortex contains abundant subpleural lymphatic vessels, and there is a network of subpleural lymphatic vessels that drain lymph from the lung surface [21]. Autopsy studies of COVID-19 patients also reported that the severity of lung lesions varied, and new and old lesions often co-existed [22, 23]. Therefore, GGO can be present in the early, middle and late stages of the disease course [11, 24, 25], which is why it has become the most common imaging sign.

The disease stage and progression of COVID-19 were evaluated using imaging stages and semiquantitative scores, which were significantly positively correlated with one another. The semiquantitative scoring system used in this study was recommended by the radiology branch of the Chongqing Medical Association, is similar to the recently reported scoring method of 5 (maximal CT score, 20 or 25) or 6 (maximal CT score, 24) lung regions on chest CT in patients with COVID-19, and was used to assess the range of infected lung tissue. These scoring methods have their own advantages and limitations. For example, the five lobar scoring system is easy to learn but is not as detailed as the 20 lung segments used in the current study. However, the latter presents with limitations that it requires the operator to have a good foundation in cross-sectional anatomy. The application of the semiquantitative CT scoring system in the rapidly developing field of artificial intelligence may promote the emergence of a relatively unified scoring standard. In some cases, RT-PCR testing is limited, and the result of the RT-PCR test can take time (hours or days). In addition, results may be false-negative. Semiquantitative scoring was positively correlated with clinical severity [26–29]. Therefore, it may assist healthcare workers to quickly classify CT-positive cases of suspected COVID-19 [30, 31] in the aforementioned cases and to choose the appropriate treatment by medical guidelines to save lives and control the outbreak. The comparatively wide range of scores (0–40) used in our study helps to refine the assessment, and thus may help clinicians to understand and identify the severity of the patients’ condition. However, it is worth noting that 12/60 (20%) patients had a negative initial chest CT scan in the current study. This may be because virus particles have not yet entered or are present in small number in the lower respiratory tract.

This study has several limitations. First, it is an exploration study based on reported autopsy results, combined with anatomic, pathologic, and radiologic analyses, rather than a strict comparison between pathological specimens and imaging, warranting further pathological studies. Second, we were only able to obtain follow-up chest CT images from a small number of patients, limiting our ability to perform dynamic observation. Third, we did not perform comparison using known semiquantitative scoring methods but shared our common scoring method. Finally, the severity and characteristics of COVID-19 vary according to race and region. Therefore, the data of only 60 Han patients in this study, with few severe cases and no death cases, cannot explain all the characteristics of the disease.

In summary, the chest CT findings of COVID-19 showed certain characteristics because of the anatomical features of the human body and pathological changes caused by the virus; semiquantitative scoring of affected lung segments may further elucidate diagnosis and assessment of disease severity. This will assist healthcare workers in diagnosing COVID-19 and assessing disease severity and will facilitate the selection of appropriate treatment options, which is important for reducing the spread of the virus, saving lives, and controlling the pandemic. Therefore, chest CT is a valuable tool for facilitating the diagnosis of COVID-19.

## Supporting information

S1 Data (SAV)
